# Competing MYB networks as switches in primary and secondary metabolism in spruce

**DOI:** 10.1186/1753-6561-5-S7-P63

**Published:** 2011-09-13

**Authors:** Claude Bomal, Isabelle Giguère, Sébastien Caron, Brian Boyle, Élise Fortin, Isabelle Duval, Armand Séguin, John MacKay

**Affiliations:** 1 Centre d’Étude de la Forêt, Université Laval, Québec (QC), G1V A06, Canada; 2 Natural Resources Canada, Canadian Forest Service, Laurentian Forestry Centre, Québec (QC), G1V A06, Canada

## Background

R2R3-MYB transcription factors are regulatory genes that have been linked to key aspects of plant development and secondary metabolism. They have been implicated in the transcriptional control of phenylpropanoid, and flavonoid metabolic pathways in model plant systems. However, in trees, knowledge is still limited about the role of R2R3-MYBs in such processes. Microarray transcript profiles (9K custom cDNA array) obtained from comparison between wild type and transgenic spruces that constitutively overexpressed *Pinus taeda* MYB1, MYB8 [1], and MYB14 [2] identified putative targets in flavonoid and phenylpropanoid metabolism.Cross-comparison of these data sets identified 70 sequences that were common to the three transgenic backgrounds: interestingly, 66 of these 70 sequences were co-expressed between PtMYB1 and PtMYB8 transgenics but produced opposite profiles with PtMYB14 transgenics. Pairwise comparisons between these transgenic data sets identified 121 additional sequences showing opposite profiles. Predicted annotations and KEGG classification showed that many of the sequences were linked to the metabolism of amino acids and carbohydrates as well as flavonoids, phenylpropanoids, and terpenoids.It was hypothesized that MYB1/MYB8 and MYB14 may be part of competing transcriptional networks for the regulation of primary metabolism and secondary metabolism. The opposite transcriptional responses might also support structural or defensive oriented response, respectively.

## Material and methods

A qPCR based experimental approach was used to test these hypotheses by evaluating transcript profile of the negatively co-expressed sequences together with MYB1, MYB8 and MYB14. Transcript levels were monitored during a diurnal cycle in three year-old wild type spruces, based on the fact that some genes associated to phenylpropanoid metabolism [3] and stress [4] were reported to follow a diurnal transcript variation. Our analysis also included a set of 10 additional MYBs putatively linked to secondary cell wall deposition [1] and stress oriented response [2]. A Spearman correlation rank test was used to estimate significant correlation (adjusted *P* val ≤ 0.01, FDR 1%) between MYB transcript profiles and selected co-expressed sequences. A functional assay system developed in embryogenic spruce cells was used to screen for possible interactions between transcription factors and promoters of putative target genes.

## Results

Among the co-expressed sequences tested, five sequences coding for 4CL, DHS2, OMT1, SMT4, and a Lipase thioesterase displayed transcript profile that positively correlated with those of PgMYB1, PgMYB8 but negatively with those of PgMYB14 and PgMYB15, the closest homologue of PgMYB14 [2]. Interestingly, these oppositions were observed in secondary xylem differentiating tissues but not in bark/phloem. Significant correlations were also observed between the co-expressed sequences tested and the others MYBs. A separate analysis of large scale expression profiles for spruce transcription factors gave some additional evidences that these putative competing MYB networks may be tissue-preferential. Transient expression results obtained with our functional assay system further suggested interactions between these MYBs and some of their proposed targets. More extensive testing is in working progress.

## Conclusions

The results of the present study strongly suggest that putative MYB networks may compete to allow rapid metabolic switch within wood forming tissue in order to direct metabolic flux towards specific aspects of primary and secondary metabolism for structural, or defensive-oriented response.
